# Developing drugs that can cross the blood-brain barrier: applications to Alzheimer's disease

**DOI:** 10.1186/1471-2202-9-S3-S2

**Published:** 2008-12-10

**Authors:** William A Banks

**Affiliations:** 1 GRECC, Veterans Affairs Medical Center-St. Louis and Saint Louis University School of Medicine, Division of Geriatrics, Department of Internal Medicine, 915 N. Grand Blvd, St. Louis, Missouri 63106, USA

## Abstract

Development of therapeutics for the central nervous system is one of the most challenging areas in drug development. This is primarily because, in addition to all of the other complications one faces in developing new drugs targeting peripheral sites, one must also negotiate the blood-brain barrier (BBB). There are dozens of strategies to overcome the obstacle of the BBB, but many of these are bound to fail, barring extreme serendipity, because they are based on an inaccurate or incomplete picture of the BBB. This article therefore starts with a brief review of the BBB as it pertains to drug development. It then examines some examples of the delivery of drugs to the central nervous system that are relevant to Alzheimer's disease, placing emphasis on peptides, antibodies, and antisense oligonucleotides.

## Introduction

This review will first examine some of the history and basic concepts of brain barriers. It will then discuss the major mechanisms that promote or retard the passage of substances from blood to brain. Finally, it will discuss specific examples of substances that cross the blood-brain barrier (BBB) and the mechanisms they most influence their abilities or inabilities to cross the BBB.

## Brief history of the blood-brain barrier

The BBB can be viewed as a concept to explain the late 19th century observation that basic dyes injected into the blood stream failed to stain central nervous system (CNS) tissues [1]. Early on, many believed that this was simply because CNS tissue had no affinity for these dyes, but another theory developed over the decades – that some barrier prevented the dye from leaving the circulation and entering the interstitial fluid of the CNS. The leading contender for this barrier was the brain's vasculature. However, gross inspection and light microscopic studies failed to show any differences between peripheral and central blood vessels. It was not until the ultrastructural studies of Karnovsky and colleagues in the late 1960s and early 1970s that the capillary bed of the brain was found to differ from peripheral capillary beds in three fundamental ways: the intercellular spaces between adjacent capillaries are obliterated by tight junctions; pinocytosis is greatly decreased; and fenestrations and other intracellular leaks are essentially absent. Together, these modifications prevent the formation of a plasma ultrafiltrate, and so plasma proteins such as albumin do not cross from blood into the CNS. Because the basic dyes bound tightly to albumin, they also were unable to enter the CNS.

Parallel barriers exist at the choroid plexus and at most of the circumventricular organs, the latter barriers formed by ependymal cells and tanycytes. Together, these barriers control the exchange of substances between blood and the CNS, but they also perform functions apart from acting as a barrier. The inability to produce a plasma ultrafiltrate means that some other mechanism must be found that conveys needed nutrients to the CNS. The barriers perform this function as well. Specific, saturable transport systems exist for the blood-to-CNS transport of glucose, amino acids, vitamins, minerals, fatty acids, electrolytes, and other substances that are needed by the CNS. Transporters oriented in the CNS-to-blood direction can rid the CNS of toxins and can act as a functional barrier to circulating substances. Small, lipid soluble substances are also able to cross the barriers, and a residual leakiness of the barrier systems (termed the extracellular pathways) can allow minute amounts of substances to enter the CNS.

Most authorities emphasize that, of these various barriers, it is the vascular barrier that is of most interest for drug delivery. This is because no CNS cell is more than about 40 μm from a capillary, and so the whole brain can be accessed by a substance delivered by way of the vascular system. Additionally, substances entering the CNS via the choroid plexus will enter the cerebrospinal fluid (CSF). These substances can distribute throughout the cranial CSF, but CSF-to-brain diffusion is limited, making penetration deep into brain potentially problematic. Finally, the vascular barrier lends itself more readily to *in vivo *and *in vitro *study and analysis than either the choroid plexus or the tanycytic barriers.

## Specific strategies for drug delivery

Dozens of strategies have been devised to deliver drugs across the BBB. Much attention has been focused on finding a universal delivery system that can carry any desired drug into the CNS. Some of these have been based on some understanding of the BBB, whereas others have disregarded essential aspects of BBB function. An alternative strategy that more closely resembles the traditional approach to drug development is as follows. Rather than starting with some universal delivery system for delivering an undefined drug (for an unknown disease), it starts with an identified ligand, usually an endogenous substance or proto-drug, targeted to a known disease. Special characteristics of the disease may aid or impede drug delivery, and the ligand can be modified to cross the BBB, which itself may be modified by the disease [2-4].

BBB drug delivery is complex because there are important exceptions to every rule and relevant caveats to every exception. Nevertheless, there are mechanisms that promote, as well as others that hinder, the passage of a substance into the CNS. Two important conclusions from this kind of analysis are: 1) the biggest barrier to delivering a substance into the CNS may not be the BBB itself, and 2) very little drug is needed in the CNS to produce a therapeutic effect. For example, only about 0.02% of a peripherally administered dose of morphine enters the brain, but that is sufficient to produce analgesia. For most CNS therapeutics on the market, less than 0.2% of the peripheral dose is taken up by brain.

There are a limited number of mechanisms by which substances cross the BBB. The major mechanisms for delivery of substances into the CNS are transmembrane diffusion and saturable transport [5]. Most CNS therapeutics are small, lipid soluble molecules that are likely to rely upon transmembrane diffusion to cross the BBB. Although peptides, and even some small proteins, have a measurable transmembrane diffusion, saturable transporters are liable to be the most effective mechanism for delivering these molecules into the CNS. Saturable transporters typically deliver 10 to 100 times more of their main ligand to the CNS than would occur with transmembrane diffusion. Substances with a small volume of distribution and a long residence time in the circulation can slowly enter the CNS by way of the extracellular pathways. Immune cells cross the BBB by a vesicular related process: diapedesis. The binding and internalization phases of this process are initiated by lectin-like interactions, that is, by interactions between glycoproteins on the endothelial surface with glycoproteins on the immune cell surface. Glycoproteins themselves can be taken up and transported across the BBB by the vesicular process of adsorptive endocytosis [6]. It may be a form of these vesicular mechanisms that the larger universal carriers are co-opting, just as some viruses exploit aspects of transport processes to cross the BBB. Two problems with utilizing diapedesis/vesicular-like mechanisms are as follows: its reliance on an intimate cross-talk between the brain endothelial cell and the immune cell, mediated largely by cytokines, and a lack of understanding of how vesicles are routed within the cell. Nevertheless, diapedesis might be an important mechanism for stem cell, immune cell, viral, and drug delivery.

There are numerous mechanisms that can oppose entry of substances into the brain. These are operable to varying degrees for a given substance. Besides the physical barrier of the endothelial cell wall, there are the following obstacles: protein binding in the circulation; enzymatic degradation in the circulation, at the BBB, or within the CNS; uptake or sequestration by peripheral tissues of the peripherally administered substance; sequestration by the capillaries that comprise the BBB; and efflux, or removal, by CNS-to-blood transporters. Countering these mechanisms can turn an ineffective drug into one that is capable of significant accumulation within the CNS.

Usually, protein binding results in a drastic net decrease in CNS uptake because only free drug is available to cross the BBB [7]. Interestingly, it was protein binding of small basic dyes that led to one of the seminal observations in describing the BBB, namely that dyes readily entered and stained the brain when perfused through brain vasculature in the absence of proteins. Protein binding can rarely assist delivery to the CNS by improving pharmacokinetics (for example, longer half-life in the circulation, smaller volume of distribution in peripheral tissues, and protection from enzymatic degradation). Unfavorable pharmacokinetics (such as short half-life, large volume of distribution, and degradation in the blood and by peripheral tissues) probably prevent as many candidate therapeutics from entering the CNS as does the physical aspect of the cell wall forming the BBB. This is especially a problem for peptides and regulatory proteins. Small, enzymatically stable peptides are capable of crossing the BBB even in the absence of a saturable transporter in amounts sufficient to affect CNS function.

Efflux transporters have emerged as a major force in drug development. They transport substances in the CNS-to-blood direction that would otherwise accumulate in the CNS. There are numerous efflux transporters, and they are known to remove many drugs, but P-glycoprotein is the most studied. Efflux of a drug can be viewed as desirable because it prevents unwanted CNS side effects (for example, loperamide and ivermectin) or undesirable because it blocks effective delivery of a therapeutic to the brain (for example, antiretroviral and anti-epileptic drugs). Efflux from the CNS of the opiate loperamide prevents it from exerting its analgesic actions but not its constipating effects on the gastrointestinal tract. Thus, loperamide is commonly used as a nonsedating treatment for diarrhea. Efflux of the anthelminthic agent ivermectin prevents it from exerting otherwise lethal neurotoxic effects. Efflux of antiretroviral drugs (for example, protease inhibitors) prevents them from effectively treating HIV-1 within the CNS, allowing virus there to replicate safely and possibly reinfect the rest of the body. Efflux of anti-epileptics is a major reason why approximately 30% of epilepsy patients are resistant to most of the currently available anticonvulsants.

## Developing drugs for Alzheimer's disease

Drugs currently on the market are traditionally small, relatively lipid-soluble compounds. Thus, these drugs can cross the BBB by means of transmembrane diffusion. With the knowledge that such a mechanism can be extended to small peptides, a series of 'breaker peptides' were developed. These substances prevent or reverse the oligomerization and fibrillation of amyloid β protein. They have been shown to cross the BBB, decreasing the presence of neurofibrillary tangles, and to reverse cognitive impairments in animal models of Alzheimer's disease (AD) [8].

Passive and active immunization have received much attention as potential treatments for AD. The main difficulty with antibodies is that they cross the BBB poorly. This poor penetration of IgG molecules is partly due to their large size, the lack of a saturable blood-to-brain transport system, and the likely presence of an effective brain-to-blood efflux transporter. The main advantages for antibodies are pharmacokinetic: they have long half-lives in blood and small volumes of distribution. Thus, they are ideal for crossing the BBB by way of the extracellular pathways. However, their accumulation in brain is abbreviated because of an apparent efflux system mentioned above. Overcoming this efflux, perhaps with the use of the IgM class, should allow enhanced accumulation of antibody in the CNS. In theory, the efflux system could work toward a therapeutic effect for an antibody with very high affinity for β amyloid protein. In this scenario, the benefit of the efflux system in helping to rid the CNS of amyloid-bound antibody would outweigh the decrease it caused in antibody accumulation within the CNS.

Saturable transport systems remain the most effective way to deliver therapeutics to the CNS. The feeding hormones are an unexpected source of peptides active in cognition. Many of these cross the BBB by way of saturable transport systems to exert their effects on the CNS. A recent example of this is ghrelin, a substance produced by the stomach, which is transported across the BBB into the hypothalamus, where it induces hunger. Ghrelin also crosses the BBB at the hippocampus, where it increases synaptic density. Ghrelin has been shown to improve learning and memory in various models, including AD [9]. Another example of using a saturable transporter is in the delivery of phosphorothioate antisense oligonucleotides to the brain. A nuclease-resistant antisense oligonucleotide directed against the β amyloid protein region of amyloid precursor protein can cross the BBB, reduce levels of β amyloid, and reverse well-established cognitive deficits in animal models of AD [10].

## Conclusion

The development of drugs that can cross the BBB is one of the greatest challenges in medicine today. However, the basic mechanisms that govern both entry to, and exclusion of, substances from the CNS are well outlined. How these mechanisms operate vary among substances and disease states, potentially adding a dimension of complexity but also of opportunity. New classes of drugs for the treatment of AD that exploit these mechanisms of BBB penetration include peptides, regulatory proteins, antibodies, and antisense oligonucleotides.

## List of abbreviations used

AD: Alzheimer's disease; BBB: blood-brain barrier; CNS: central nervous system; CSF: cerebrospinal fluid.

## Competing interests

The author received a small grant for Serono to study the permeability of the BBB to breaker peptides. The author is a shareholder and scientific consultant for EDUNN, a biotech company investigating the development of antisense molecules for the treatment of Alzheimer's and other CNS diseases.
